# A Novel Primary Care Planning Informatics Tool Informed by Data-Driven Multimorbidity Grouping: User-Centered Design and Feasibility Testing

**DOI:** 10.2196/75081

**Published:** 2025-12-04

**Authors:** Linnaea Schuttner, Karin Daniels, Srinidhi Venkatesh, Franya Hutchins, Karley Atchison, Rebecca Piegari, Timothy Bober, Xinhua Zhao, Ann-Marie Rosland

**Affiliations:** 1 Health Services Research & Development, VA Puget Sound Health Care System, 1660 S Columbian Way, Seattle, WA, 98108, United States, 1 2062776126; 2 School of Medicine, University of Washington, Seattle, WA, United States; 3 VA Center for Health Equity Research and Promotion (CHERP), VA Pittsburgh Healthcare System, Pittsburgh, PA, United States; 4 Department of Medicine and Caring for Complex Chronic Conditions Research Center, School of Medicine, University of Pittsburgh, Pittsburgh, PA, United States; 5 Department of General Internal Medicine, Pearlman School of Medicine, University of Pennsylvania, Philadelphia, PA, United States; 6 Analytics and Performance Integration, Veterans Health Administration, Washington, DC, United States

**Keywords:** complex care, panel management, population health, primary care, multimorbidity, care planning

## Abstract

**Background:**

Patients with multimorbidity have complex health care needs and are at high risk for adverse health outcomes. Primary care teams need tools to effectively and proactively plan care for these patients. We developed VET-PATHS (Veteran Panel Management Tool for High-Risk Subgroups), a novel care planning informatics tool for complex primary care patients. VET-PATHS groups patients by chronic condition via latent class analysis of electronic health record data, then jump-starts care planning by suggesting “care steps” based on data-driven high-priority care for the group.

**Objective:**

The study aimed to iteratively adapt VET-PATHS with user input, then test feasibility and acceptability by frontline primary care teams for empaneled patients at high risk.

**Methods:**

Three rounds of user-centered design sessions with 17 primary care providers and registered nurses at 5 sites from 2019 to 2021 were conducted to obtain feedback on the VET-PATHS layout, content, and user interface. Feedback was summarized into 4 user experience domains (useful, desirable, credible, and usable), leading to progressively updated prototypes. After the national tool release, we conducted a pilot intervention study in 2023‐2024 with 6 primary care teams at 4 sites using VET-PATHS during asynchronous regular meetings. Tool use and resulting care plans were assessed by templated observation during meetings, postpilot chart review, and administrative data. Individual qualitative interviews were analyzed by rapid template analysis for feasibility, acceptability, and utility.

**Results:**

User-centered feedback led to updated tool content, context (eg, use in proactive panel management), targeted users (eg, focusing on primary care providers), and display layout. Pilot teams used VET-PATHS over 4 to 8 weekly meetings (mean length 24, range 16‐49 min), actively reviewing 80% (280/351) of empaneled patients at high risk visible in the tool. Tool use prompted 127 new actions for 91 unique patients (33% of patients reviewed) and documentation of >1 new care plan for 19% of patients reviewed. Common actions included requests to return to the clinic (n=34, 27%), referrals (n=25, 20%), or vaccinations (n=24, 19%). Of the 127 actions planned, 53 (42%) were received by patients. Difference-in-difference trends for acute hospitalizations declined post pilot, while outpatient utilization was stable or increased for pilot team patients compared to all patients at high risk at pilot sites (per-patient counts: acute hospitalizations −0.15; primary care visits 0.00; mental health visits 0.99). Four generalist teams (n=11 interviews) described higher acceptability. Two “focused” teams with more homogenous panels, for example, substance use disorder (n=3 interviews), found care steps less useful. Teams described how VET-PATHS improved efficiency of care planning through automated patient grouping and identification of care gaps and increased multidisciplinary involvement.

**Conclusions:**

User-centered improvements to VET-PATHS were designed to help clinicians process and use complex information about patient multimorbidity to efficiently create new care plans. Subsequently, VET-PATHS was acceptable and feasible to frontline primary care teams, particularly with more general patient panels, and led to concrete changes to clinical care delivery.

## Introduction

The number of patients with multimorbidity (ie, multiple chronic health conditions) and complex health needs is increasing as chronic diseases accumulate at younger ages [1,2]. In the Veterans Health Administration (VHA) and other health care systems, the top 10% of patients who are most complex account for 80% of health care utilization [3]. Patients with multimorbidity are also at high risk for worse health outcomes, including functional decline [4,5] and death [6]. These patients require greater time and effort from health care staff in primary care visits [4,7,8], potentially adding to clinician burnout [9,10]. Delivering care that effectively improves health outcomes for these patients is an ongoing but important challenge requiring innovative solutions.

Many health care systems, including VHA, have risk-prediction scores that identify patients at high risk for hospitalization or other adverse events; however, clinicians are left wondering what to do once a patient is identified as high risk [11]. “One-size-fits-all” interventions for high-risk patients have not been successful [12,13], in part due to heterogeneity in concurrent multimorbidity. In VHA, primary care teams have access to tools that highlight which of their patients are at high risk but have described feeling overwhelmed by the volume of patient health information to review and the lack of guidance on what care needs are most important for high-risk patients [14-16]. Other tools are focused on specific health needs or diseases, such as preventive care, to identify care gaps [17]. However, to our knowledge, no tools within or outside VHA currently exist that identify and prioritize care needs across health conditions for high-risk patients with complex multimorbidity.

One promising approach is to develop individualized care plans for patients with multimorbidity. Care planning involves a comprehensive review of patient health and care history and the development of a multidisciplinary action plan [18]. Care planning has shown promising results in specialized settings (eg, geriatrics) and populations (eg, diabetes) [19-23] and is widely recommended for high-risk patients and those with complex multimorbidity [13,24,25]. However, individualized care planning has not been successfully implemented in primary care due to the amount of time, effort, and resources required for this patient population, as well as varied care needs [12,23,26].

Population segmentation for primary care patients with multimorbidity could help bridge the gap between time-consuming individualized care planning and ineffective one-size-fits-all interventions. Population segmentation is a popular analytic technique used by health care systems to identify groups of patients more similar to each other within the wider patient population [27-30]. Data-driven segmentation applied to patients with complex multimorbidity could identify groups of patients for tailored, group-specific interventions. To date, population segmentation has not been bridged beyond observational uses to identify patients for health care interventions within the care delivery of high-risk patients.

In this paper, we describe the user-centered design and pragmatic pilot testing of VET-PATHS (Veteran Panel Management Tool for High-Risk Subgroups), a novel clinical informatics tool to facilitate care planning for high-risk primary care patients with multimorbidity. VET-PATHS uses population segmentation to sort patients with high-risk scores for hospitalization into groups of patients with similar patterns of multimorbidity and identify priority care gaps based on group-specific risks for hospitalization and a patient’s prior receipt of care. In this study, frontline primary care teams gave input into the VET-PATHS design, then used VET-PATHS to generate care plans for their empaneled patients who were at high risk, tailored to multimorbidity groupings and individualized patient needs. Our study objective was to describe the user-centered design process and results of a feasibility and acceptability pilot of VET-PATHS for team-based management of patients at high risk with multimorbidity within the VHA.

## Methods

### Primary Care Setting

In the VHA, primary care is delivered to over 6 million veterans in over 1100 facilities through multidisciplinary care teams (*Patient Aligned Care Teams* [PACTs]) [31]. Fully staffed PACTs include a primary care clinician (PCP), registered nurse (RN) care manager, clinical associate (CA; eg, licensed vocational nurse), and medical support administrator (MSA) at a ratio of 1 full-time equivalent PCP to 3 non-PCP “core” PACT members. Patients are empaneled to a specific PACT at a ratio of 1200 patients per 1.0 full-time equivalent PCP. “Extended” PACT multidisciplinary providers are shared across core PACTs and include embedded clinical pharmacists, social workers, mental health providers, and nutritionists. PACTs are encouraged to use “panel management” tools during asynchronous (nonpatient time) team meetings to identify and meet health needs for subpopulations of patients on their panel, such as identifying and contacting all empaneled patients currently due for cancer screening [17].

### VET-PATHS Patient Population

VET-PATHS displays a team’s empaneled primary care patients with a high VHA Care Assessment Need (CAN) score. The CAN score is a validated risk prediction score for adverse outcomes used nationally in VHA [32]. Patients are included in VET-PATHS if they have a CAN score ≥90th percentile at any time within the past 1 year, corresponding to an approximate 25% risk of hospitalization; patients with newly high CAN scores are added quarterly. Once included in VET-PATHS, patients remain visible within the tool for 12 months after their most recent qualifying CAN score.

### Tool Description

VET-PATHS is available on a VHA intranet website alongside other panel management tools and population health registries. The tool draws from databases of electronic health records (EHRs) to display patients actively empaneled with the team and the patients’ up-to-date medical and health care information. Patients are first displayed in a panel view, sortable by multimorbidity group label (described later) and key demographic and clinical information. A second individual patient-level view provides suggested care plan elements (“care steps,” described below) tailored to the patient’s group, alongside additional relevant patient health and utilization information (see Multimedia Appendix 1 for tool screenshots).

1. Multimorbidity groups: Patient assignment to multimorbidity groups is derived from previously described and validated population segmentation [33-36]. Briefly, these models identify latent multimorbidity groups among high-risk Veterans using 31 chronic conditions [36] identified via health care encounters within the prior 24 months, based on *International Classification of Diseases, 10th Edition* codes from the US Department of Health and Human Services’ multiple chronic conditions framework [37] and expanded for Veteran care (eg, inclusion of posttraumatic stress disorder; Multimedia Appendix 2). At the time of the pilot intervention reported here, there were 6 multimorbidity groups used in the tool, named for the most common diagnoses among patients assigned to that group: cancer, cardiometabolic, liver, mental health, substance use, and low comorbidity. Veterans are uniquely assigned to a group if the models show a probability of matching ≥70% to the group; patients are not required to have all of the group conditions to be assigned and often have differing but overlapping sets of conditions. Around 6% to 8% of patients at high risk do not meet the threshold for assignment to any group and are labeled “unassigned.”
2. Care steps: VET-PATHS also provides suggested care plan components for the primary care team to consider for each patient. These are called “care steps” in the tool and are tailored to each multimorbidity group’s unique needs. Within each group, the individual patient-level view displays each care step as (1) applicable to the patient but not yet received (*due*); (2) applicable and already received, with date of receipt (*done*); or (3) not applicable to the individual patient (*N/A*). Care step status is automatically drawn from administrative databases linked to the patient’s EHR for care already received (including encounters, diagnoses, and laboratory results) but is not able to capture data from “free-text” EHR notes. Care step status based on receipt of health care is updated daily. Care steps are presented if relevant to the patient’s multimorbidity group; an individual patient may not have all steps “due” or “applicable” and may have other individual care needs not part of the group’s care steps and thus not displayed by the tool.
3. Steps are presented within the tool as optional suggestions. The need for clinician review and agreement was emphasized in tool training, graphics, and documentation. During the pilot, 4 of 6 multimorbidity groups had care steps programmed and available for preliminary testing and were the focus of the pilot intervention (cardiometabolic, liver, mental health, and substance use groups).

Care steps target three main types of care plan components for each group. First, some care steps target common gaps in quality metrics for a multimorbidity group. For example, a care step may suggest hemoglobin A_1C_ testing in patients with diabetes, if due, based on prominent group-level gaps in this metric for mental health group patients. Other care steps highlight common gaps in health care services within the group in response to adverse medical events [34], for example, screening for fall risk factors in groups with comparatively high rates of medically documented falls or nephrology referral in groups with a frequent diagnosis of advanced kidney disease but underuse of this guideline-recommended service. Third, care steps address unique risk factors for hospitalization identified for each group based on their EHR data [34], for example, addressing alcohol use above recommended limits for groups with comparatively higher hospitalization risk in patients with alcohol use disorder.

We initially developed and iteratively refined 3 to 5 care steps for each group within VET-PATHS. Initial candidate care steps were chosen based on gaps in care identified from observed and predicted differences in clinical conditions, utilization patterns, and health outcomes specific to each group [34,38]. Specific care steps were aligned with relevant VHA/Department of Defense Clinical Practice Guidelines when applicable. Care step candidates then went through three rounds of clinical expert review: two multispecialty clinical panels conducted with 39 unique experts representing VHA program offices (eg, Primary Care and Population Health), clinical specialties (eg, cardiology, substance use disorder, geriatrics), and special populations (homeless care, women’s health); then input from frontline clinicians during preliminary testing. Care steps were designed to not overlap with existing population health management, registry tools, or clinical “reminders” in the EHRs; although developed for VET-PATHS prior to release as a clinical reminder, one care step (hepatitis B vaccination) for liver and substance use group patients was available to some sites as a VHA national clinical reminder released in October 2018 (for at-risk adults, universal screening was recommended in 2023 [39]).

### Iterative User-Centered Design and Prototype Development

From 2019 to 2021, we conducted three rounds of user-centered design to develop the VET-PATHS content and user interface [40]. Target primary care clinical users were recruited via staff meetings and purposeful outreach to clinicians already serving as champions in related projects. Eligible users were either PCPs or RN care managers within an active primary care role at a VHA site; RNs and PCPs were recruited separately, not as team dyads. For each round, we conducted 20‐ to 45-minute virtual or in-person individual semistructured interviews. Prototypes were updated based on interviewee input between each round. Round one (2019) used paper and static screen mock-ups, displaying fictional patient data to interview participants. Participants were asked about the interpretation of displayed clinical information, design features, and perceived usefulness. Round two (2019) was completed using electronic visuals (screenshots, pictures) of an updated prototype completed with simulated patient information. Participants were asked in this phase about clinical interpretation, design features, and potential workflows for tool use. Round three (2021) used an interactive web-based version of the tool. This version displayed the clinician’s actual patient panel and patients’ real-time health information. In this round, participants were asked to “walk through” how they would navigate, interpret, and use different parts of the tool and describe how the content related to the care or understanding of their patients.

Interviews and walk-throughs were audio-recorded and conducted by a PhD-level staff member, with a research coordinator taking detailed notes verified via recording review for accuracy and completeness. Feedback was summarized into deductive categories guided by 4 user experience domains (useful, desirable, credible, and usable) [41], as well as validation of the tool content for real-world patient care (round 3 only). Updates based on the summary reports were made to the tool between each design round, and user interview guides were refined in response to results and Veteran stakeholder board (2019) feedback. Following these three rounds of user-centered improvements, the tool was released for nationwide clinical use in 2021.

### Intervention Pilot Study

In 2023‐2024, we conducted a pilot intervention study with frontline PACTs to examine the feasibility, acceptability, and perceived utility of using the tool to develop care plans for empaneled patients with complex multimorbidity. Outreach to potentially interested teams was based on recommendations by site leadership and peer referral. After a clinician “champion” from a team agreed to participate, the team was trained on tool use. Study facilitators then scheduled 30-minute weekly or biweekly meetings, during which the team met to review and discuss their patients displayed in VET-PATHS by multimorbidity group, for a maximum of 8 weeks. Champions could include any core or extended PACT members in their meetings at their discretion. Teams were provided with optional adaptable documentation templates to track care planned for a patient in the main EHR. Study facilitators supported team meetings, including answering technological questions if asked (eg, how to navigate screens within the tool), but were instructed not to provide unsolicited prompts on tool use, advise teams on clinical interpretation or application of information, or otherwise direct how teams used the tool for patient care. Two study facilitators attended each meeting for data adjudication and fidelity review.

Data on tool use and resulting clinical activity were collected from multiple sources. First, study facilitators directly observed team discussions and actions intended during their meetings, recording observations into a structured data collection template. Second, manual chart reviews were completed by separate, nonfacilitator study staff approximately 60 days after the team’s final meeting. For both facilitator observation and chart review, actions considered related to VET-PATHS meetings, which included (1) tool-suggested care steps discussed and related intended actions (eg, based on VET-PATHS care steps, team decided a patient should receive a vaccine; PCP stated that a patient should get updated laboratory tests); (2) new individualized care plans documented in the EHR after the team meeting tracking intended actions, using a recommended “Care Plan” note template or note addendum; (3) intended actions carried out or ordered (eg, phone call by nurse to discuss vaccination documented; laboratory order placed in the EHR by the PCP); and (4) actions received (eg, patient actually received the vaccine; patient completed laboratory test). Following the chart review for data collection, two other study members (MD and PhD level) completed data abstraction for clinical activity independently, using prespecified case definitions. Agreement between abstractors was 92%, with remaining differences resolved by discussion. Third, at the conclusion of the 8-week pilot period, team members were sent an electronic confidential System Usability Scale (SUS) [42], adapted to the VET-PATHS tool and its functions. SUS questions are scored on a 1 to 5 Likert scale, with a maximum possible total of 100 points (higher is more usable); a score of 68.0 (industry average) or higher is considered “acceptable” usability [42,43]. Fourth, qualitative baseline interviews with the PACT champion and postintervention individual interviews with each participating team member were conducted by trained study staff. Postintervention interviews were conducted by staff uninvolved in the pilot or facilitation. Interviews were audio-recorded and transcribed using Microsoft Teams; then, transcripts were verified by another study team member. Interview topics included contextual aspects (eg, typical patient care and panel management workflows, usual team workload division, staffing shortages) that informed prepilot team training and meeting logistics and other factors that influenced team acceptability [44] and usability of the tool (eg, competing clinic initiatives) in both pre- and postpilot interviews. Qualitative data were analyzed by team and by clinical role. Two study qualitative analysts not involved in the user-design testing or pilot intervention completed rapid template analysis of transcripts [45], with result interpretation supported by discussion and consensus among the larger study team. Finally, administrative data outcomes for utilization were collected as exploratory findings. This pilot study was not designed with the power to detect statistically significant differences in outcomes; therefore, we limit exploratory outcome analyses to descriptive statistics only [46,47]. Patient baseline outcomes for 3 months prior to the index date of team enrollment were compared within 3 to 6 months post final team meeting, using an intention-to-treat difference-in-difference approach. Comparator trends were for similar time periods among patients with high CAN scores within each pilot site but not enrolled on a pilot team.

### Ethical Considerations

This work was carried out as a quality improvement evaluation and designated nonresearch under the terms of a signed attestation from the VHA Office of Primary Care, which exempts this work from institutional review board review, in accordance with the national VHA Office of Research and Development’s nonresearch policy under the US Department of Veterans Affairs. This VHA documentation and review process ensures that work not carried out under a human subject’s protocol is part of institutionally sanctioned quality improvement activities. Primary participant data (eg, qualitative interviews) specific to project activities are collected with individual verbal informed consent and handled in accordance with ethical and privacy guidelines consistent with research-designated data. Data from participants were deidentified for analysis and anonymized for reporting. No additional compensation was provided to individual participants outside of their usual salary; however, reimbursement of time for team participation in pilot testing was covered by the project to offset patient care time needed for quality improvement activities.

## Results

### User-Centered Design (Prototype Development) Phase

A total of 17 clinical users (Tables1  2), including PCPs (n=5) and RN or nursing supervisors (n=12), completed interviews at 5 VHA primary care sites across the United States (Northeast: n=8; Midwest: n=3; South: n=4; and West: n=2). Round one of user interviews (n=6 RN, paper prototype) led to refining the tool and workflows to target PCPs for care recommendations within care steps (usefulness). Users felt the tool was desirable for some contexts, including perceptions that using the tool would be helpful for proactive panel management, improving understanding of new patients to a provider, and identifying patients in need of unscheduled proactive outreach to prevent missed care and health decline, and avoid reactive and acute care. Tool credibility was perceived as boosted by specific factors, including integration into a currently used portal and use of established diagnosis and risk-prediction scores. Round two (n=2 PCPs, n=6 RNs, electronic prototype) revealed user perception of the tool as useful in specific contexts: panel management for a group of patients, providing feedback to a patient on why care steps might be recommended, or in preparation for a patient visit. Tool content was seen as desirable if information did not overlap with existing tools, the tool was integrated with the EHR, and the care steps were nonredundant, which current “clinical reminders” staff were already asked to complete during encounters. Credibility was felt to be enhanced by regular and visible updates. In round three of interviews (n=3 PCPs, web-based interactive prototype), users were interviewed as they “walked-through” the tool. They perceived usefulness as related to the type of information displayed and integration with the EHR. The care steps were perceived as desirable to cover gaps in care not previously considered for the patients. Credibility was felt to be related to the transparency of the data origins underlying the care steps. All three rounds led to updates made to the tool content, format, graphics, and text (see Multimedia Appendix 3 for overview of changes, such as views and content updates, terminology revisions, and formatting changes).

**Table 1.** Participant characteristics in VET-PATHS^a^ development.

Round and role		Type of prototype test	Site/region	
User-centered prototype development, n=17				
Round 1 (6 nurses; RN^b^)		Paper prototype	Urban VAMC^c^ (Northeast)	
Round 2 (2 PCPs^d^)		Paper prototype	Urban VAMC (Northeast)	
Round 2 (6 nurses; RN)		Screenshot visuals with simulated patient data	2 urban VAMCs (Midwest, South)	
Round 3 (3 PCPs)		Web-based interactive prototype with actual patient data	2 urban VAMCs (South, West)	

a VET-PATHS: Veteran Panel Management Tool for High-Risk Subgroups.

b RN: registered nurse.

c VAMC: Veterans Health Administration medical center–affiliated clinic.

d PCP: primary care clinician.

**Table 2.** Participant characteristics in VET-PATHS^a^ pilot testing.

Teams^b^	Patient panel size	Site/region
Feasibility pilot intervention (n=23; 6 teams)		
General, median (IQR)	342 (192-573)	
A, n	476	Urban VAMC^c^ (Northeast)
B, n	862	Rural CBOC^d^ (Northeast)
C, n	143	Urban VAMC (Western)
D, n	208	Rural CBOC (Western)
Focused, median (IQR)	108 (105-110)	
E, n	112	Urban VAMC (Northeast)
F, n	103	Urban VAMC (Northeast)

a VET-PATHS: Veteran Panel Management Tool for High-Risk Subgroups.

b General primary care teams care for heterogeneous patient panels. Focused teams provide medical or topic-specific care to more homogenous patients (eg, patients living with substance use disorders).

c VAMC: Veterans Health Administration medical center–affiliated clinic.

d CBOC: community-based outpatient clinic.

### Feasibility Pilot Intervention

Six teams (23 clinical staff) participated in the pilot testing of VET-PATHS within real-world clinical settings at 4 VHA sites (2 community clinics and 2 VHA medical centers) across two US regions (Tables1  2). Four teams represented typical PACTs in the VHA, providing general primary care to larger panels (median 342, IQR 192-573 total patients per team, 15% of panel appearing as high-risk in VET-PATHS). Two teams provided primary care focused on patient populations with specific medical conditions and had smaller patient panels (median 108, IQR 105-110 total patients, 56% of panel as high-risk within VET-PATHS). Teams conducted between 4 and 8 meetings each across an 8-week period. The mean length of meetings was 24 (range 16‐49) minutes. A range of multidisciplinary roles attended meetings, with PCP Champions attending all meetings. General teams had attendance at most meetings from “core” team members (RN, CA, and MSA), while focused teams more often had other extended PACT members attend (Table 3).

**Table 3.** VET-PATHS^a^ feasibility results among 6 pilot teams (4 general and 2 focused).

	General					Focused		
	Team A	Team B	Team C	Team D	Mean (SD)	Team E	Team F	Mean (SD)
Site	Urban VAMC^b^	Rural CBOC^c^	Urban VAMC	Rural CBOC	—^d^	Urban VAMC	Urban VAMC	—
Total team panel size, n	476	862	150	411	475 (294)	112	103	108 (6)
HR^e^ patients in tool, n	88	91	24	27	58 (32)	60^f^	61	61 (0.5)
HR patients reviewed in meetings, n/N (%)	77/88 (88)	87/91 (96)	23/24 (96)	27/27 (100)	94.7 (4.5)	39/60 (65)	27/61 (44)	54.6 (10.4)
Team meetings, n	8	6	6	5	6.3 (1.1)	6	4	5.0 (1.0)
Time reviewing patients per meeting (min)	21	49	21	17	27.0 (12.8)	16	19	17.6 (1.4)
Meeting attendance by role (%)	100% PCP^g^^,^^h^ 13% RN^i^, 88% MSA^j^^,^^h^	100% PCP^h^, 100% RN^h^, 50% CA^k^, 100% MSA^h^	100% PCP^h^, 83% RN, 83% CA^h^, 67% MSA^h^	100% PCP^h^, 100% RN^f^, 100% CA, 80% SW^l^^,^^h^	74% core, 20% not core	100% PCP^h^, 60% Psych^m^, 40% Pharm^n^, 40% SW	100% PCP^h^, 100% Pharm, 100% RN, 100% CA^h^	50% core, 40% not core
Care steps “due” and discussed, n	130	165	36	41	93 (55.9)	43	36	39.5 (3.5)
Total new actions intended, n/N (%)	40/130 (31)	47/165 (28)	21/36 (58)	8/41 (10)	29.5 (16.0)	11/43 (26)	0/36 (0)	—
Of above, additional actions (not care steps), n	12	14	6	1	8.3 (5.6)	3	0	*—*
Care plans documented, n/N (%)	17/77 (22)	22/87 (25)	11/23 (39)	4/27 (15)	52/214 (24)	2/39 (5)	0/27 (0)	2/66 (3)

a VET-PATHS: Veteran Panel Management Tool for High-Risk Subgroups.

b VAMC: Veterans Health Administration medical center–affiliated clinic.

c CBOC: community-based outpatient clinic.

d Not applicable.

e HR: high-risk; predicted ≥90th percentile of risk for hospitalization within 1 year.

f Number shown in tool exceeds high-risk patients at baseline due to update in patients at the time of first pilot team meeting, after baseline demographic data were pulled.

g PCP: primary care clinician.

h Provided posttest qualitative interview.

i RN: registered nurse.

j MSA: medical support administrator (administrative clerk).

k CA: clinical associate (eg, licensed practical nurse).

l SW: social worker.

m Psych: mental health provider (eg, team clinical psychologist or psychiatrist).

n Pharm: pharmacist.

**Figure 1.**
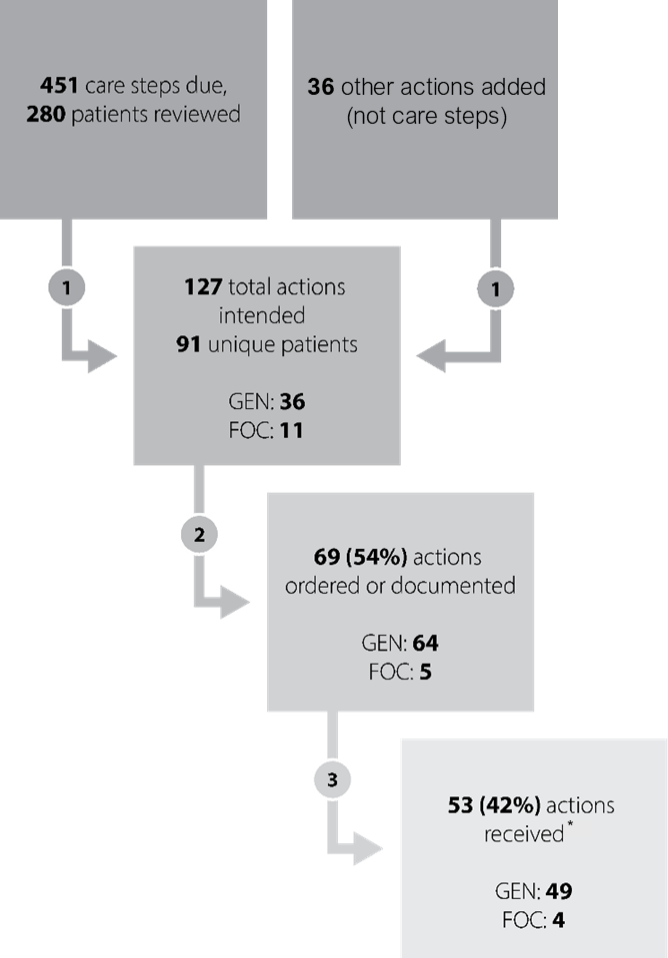
Care steps and actions received by patients during the pilot test. *Twelve of 53 (23%) did not show or declined. FOC: focused team; GEN: general team; MH: mental health.

Teams actively reviewed 80% (280/351) of the patients visible within the VET-PATHS, with more patients reviewed by general teams (214/230, 93%) than focused teams (66/121, 55%; Table 3). A total of 1343 care steps were possible for the 351 patients within 4 multimorbidity groups, and 596 (44%) care steps were “due” care suggestions for patients. For the 280 patients reviewed, 451 care steps were “due” and discussed by the team. Teams verbally discussed and intended 127 actions and documented these actions in 76 new care plans (plans were counted once only, per patient) in the EHR among patients reviewed following a team meeting. Of actions, most (91/127, 72%) were based on care steps recommended within the tool; the remainder (36/127, 28%) were additional actions that the teams added after reviewing the patient’s information in the tool, for example, a team decision to schedule a return visit for patients not seen recently in primary care. Of 127 actions planned, 69 (54%) actions led to an order placed or the team documenting an action to provide the care to the patient, and 53 (42%) actions were documented as either received (41/53) or declined (12/53) by the patient (Figure 1). Of 127 actions planned, these included return to clinic orders (34/127, 27%), referrals other than to mental health (25/127, 20%), vaccinations (24/127, 19%), other actions (21/127, 16%), referrals to mental health (16/127, 13%), and laboratory orders (7/127, 5%).

Of 280 patients discussed, actions were intended for 91 (32%) unique patients, ordered on 56 (20%), and received by 45 (16%). Of specific care steps within the tool, intended uptake (team discussion with planned action on suggestions from VET-PATHS care steps) varied by multimorbidity group and care step. Intended uptake for each group’s care steps ranged from a mean proportion of 11% (12/109 actions intended “due” and discussed by team, Liver Group) to 37% (30/82 actions intended “due” and discussed by team, Substance Use Group), with greater uptake of items with less effort to deliver, such as vaccination or laboratory orders, and less uptake of items requiring more complex decision-making or discussion with patients, such as referrals to palliative care or specialists. When teams opted to take no action on care steps “due” for patients discussed (n=363 care steps), the most common reasons stated in the team discussion were PCPs’ judgment that a care step was not a priority for the patient (92/363, 25%) or the patient already was scheduled for care that had not yet occurred (42/363, 12%; Multimedia Appendix 4).

Tool usability surveys were completed by 15 of 19 (79%) participating users. The mean SUS score was 69.9 (SD 7.8). Usability was scored similarly between general and focused teams (general: mean 69.3, SD 7.9; focused: mean 71.3, SD 8.5).

Utilization was examined among patients at high risk on pilot teams compared to nonpilot patients at high risk at the same clinic during similar time periods. Trends in hospitalization, 30-day readmissions, and urgent care use were lower in pilot patients 3 to 6 months post intervention, with increases in primary care (telephone), mental health, and specialty outpatient use (Table 4).

**Table 4.** Difference-in-difference (DID) utilization outcomes, high-risk usual care versus VET-PATHS^a^ pilot patients.

	Usual care (n=12,230)^b^		VET-PATHS (n=341)^c^		DID: VET-PATHS vs usual care
	Pre (0‐3 m)	Post (3‐6 m)	Pre (0‐3 m)	Post (3‐6 m)	
Acute hospitalizations	1.4 (0.8)	1.4 (0.9)	1.8 (1.2)	1.6 (1.0)	−0.15
Readmissions (30-d)	1.5 (1.0)	1.5 (1.2)	1.7 (0.9)	1.6 (0.8)	−0.08
ED^d^ visits	1.6 (3.2)	1.5 (3.4)	2.0 (3.61)	2.1 (3.5)	0.18
Urgent care visits	0.04 (0.3)	0.05 (0.4)	0.04 (0.2)	0.03 (0.4)	−0.02
Primary care, in-person visits	1.5 (1.8)	1.4 (1.6)	1.4 (1.8)	1.3 (2.1)	0.00
Primary care, phone visits	1.2 (1.8)	1.1 (1.8)	0.9 (1.6)	1.4 (1.7)	0.56
Mental health, all clinic visits	8.4 (14.3)	7.3 (13.2)	7.8 (11.4)	7.8 (15.6)	0.99
Specialty care, all clinic visits	4.9 (4.6)	4.3 (4.4)	5.5 (5.9)	5.5 (5.3)	0.57

a VET-PATHS: Veteran Panel Management Tool for High-Risk Subgroups.

b Mean age 70.1 (SD 12.9) y; 69% (8239/12,002) White; 8% (935/12,230) female.

c Mean age 68.3 (SD 13.7) y; 67% (225/336) White; 12% (41/341) female.

d ED: emergency department.

Experiences with the tool and use in team meetings were described by 14 team members within posttest interviews (Table 4). All teams contributed at least one postpilot interview, and all team roles were represented (interview participants are noted in Table 3). Three main themes were identified. First, participants described efficiency related to the tool’s grouping of patients:

*This was new for me and my team to have a way to characterize groups further and have these buckets to think about. […] It helped me get to know some of my patients*

[PCP, general team]

Second, teams described the tool, making them aware of care “gaps” that had been previously hard to identify:

*it focused our efforts on certain aspects of their care that […] maybe we have neglected*

[PCP, general team]

Third, participants described VET-PATHS as enabling the whole team’s involvement in patient care planning:

*it definitely helped us to work together as a team to sort through some of our higher risk panel […] to reevaluate some of our Veterans a little bit more thoroughly*

[RN, general team]

When examined by teams and roles, general teams overall described high acceptability of the tool content and mixed acceptance of care steps, depending on the type of care step and group. They desired more automatic integration with the primary EHR, for example, one-click placement of orders recommended by care steps. Focused teams reported lower usability of the tool for their more specific patient needs and often felt they had already addressed or did not find the care steps suggested appropriate for their specific patient panels. Acceptability of the tool and specific care steps also varied across roles and teams (Tables5  6). Team members newer to panel management, less familiar with their patients, or serving more heterogeneous patients perceived more utility than those with more established panel management processes or well-known or more homogeneous patient panels. Among roles, PCPs and RNs reported overall high acceptability of the tool, with positive perceptions of the patient grouping functions and particular support for advancing the multidisciplinary team member contributions to care planning. MSAs and CAs described the acceptability of the tool as they perceived it facilitating their more support-based role to the PCP.

**Table 5.** Postpilot qualitative interview results by role.

Role	Themes	Example quotes
PCP^a^ (n=6)	Helpful to have groups to characterize patients; positive about team-based discussion. Some mixed acceptability of care steps	*Maybe I’m thinking their problems, their core issues lie here. But according to VET-PATHS profiling, they’re more of “this flavor,” right? And that was helpful to crosscheck […] my characterization of certain patients and their risk.* [PCP GEN^b^]
RN^c^ (n=2)	Helpful for organizational process (sorting, tracking) and exposing tasks not previously considered for patients. Both described processes improving team collaboration	*Having the high-risk patients there and what to consider approaching, it’s almost like a checklist, like “did we do this, did we do that?” It’s nice to have.* [RN FOC^d^]
CA^e^ (n=2)	Acceptable if used in conjunction with PCP, to reduce PCP burden	*As the [CA], it falls under my category…to get the provider all the information that she needs at a glance to make her job easier.* [CA FOC]
MSA^f^ (n=3)	Acceptable if in conjunction with PCP. Tool increased understanding of patient clinical needs, helped clerks prioritize their tasks according to clinical urgency, which they often did not have insight into	*It’s a very helpful… because for us, we schedule the patients and we were getting informed and that the patients’ needs are our priorities.* [MSA GEN]
Other (n=1)	Improved ability of team to collaboratively discuss patients	*Was helpful to go over the high-risk patients as a team… it’s good to have a list just so nobody falls through the cracks.* [SW^g^ FOC]

a PCP: primary care clinicians (MD, nurse practitioner).

b GEN: general team.

c RN: registered nurse.

d FOC: focused team.

e CA: clinical associate (eg, licensed vocational nurse, care technician).

f MSA: medical support administrator (administrative clerk).

g SW: social worker.

**Table 6.** Postpilot qualitative interview results by team.

Team	Contextual factors	Acceptability/utility	Quotes
Team A (n=2)	General team, understaffed. Non-PCP^a^ staff turnover during pilot No prior panel management process	Positive in helping identify most at-risk patients and highlighting need; some concerns about documenting care step assignment/tracking.	*I think it was a nice tool to pull out who are my high-risk patients. I think it helped me identify who had not been seen in some time [.]. [One care step] for a lot of my patients did not seem appropriate [.] but put it in the forefront.* [PCP GEN^b^]
Team B (n=3)	General team, fully staffed No prior panel management process	Positive about tool content (especially subgrouping), overall felt context and navigation were acceptable; positive reflection on increase in interdisciplinary discussion.	*It was helpful in bringing the team together and focusing on Veterans who are medically and psychosocially complicated.* [PCP GEN] *It broke out very straightforward on the things that we need to concentrate on.* [MSA^c^ GEN]
Team C (n=3)	General team, fully staffed. Recently inherited ~50% of high-risk patients Significant panel management experience	Positive about tool, felt it was useful for both subgroups and care steps. Some challenges with administrative implementation of care steps (eg, reaching patients post-meeting).	*Having sort of clear buckets and care steps that are evidence based or evidence informed, I think was really innovative and that’s [what] I’m very excited about.* [PCP GEN]
Team D (n=3)	General team, understaffed. Significant prior experience in panel management	Positive about tool, especially subgrouping and increasing interdisciplinary discussion. Felt care steps less useful, primarily due to feeling these were not applicable or already done for patients.	*It kind of validated that we were actually on the right focus. Not necessarily that it made us. Like we were already doing it. I felt like it matched what we were doing with the for the patients.* [RN^d^ FOC^e^]
Team E (n=1)	Focused team for specific care population, understaffed Doing regular previsit huddles, but not full team meetings	Some concerns about applicability of care steps to more specific population, many of whom already received suggested care.	*It’s not that helpful to see a list of your sickest Veterans and then have them just check off all of the boxes.* [PCP FOC]
Team F (n=2)	Already doing multidisc. team meetings, but not with panel tools Focused team for specific care population, understaffed	Some concerns about utility of care steps for more specific population, many of whom already received suggested care.	*The care steps that [were] listed on there, those are things that we’d already done, and we needed to go beyond those initial care steps to what’s next.* [CA^f^ FOC]

a PCP: primary care clinicians.

b GEN: general team.

c MSA: medical support administrator (administrative clerk).

d RN: registered nurse.

e FOC: focused team.

f CA: clinical associate (eg, licensed vocational nurse, care technician).

## Discussion

### Principal Findings

VET-PATHS is an innovative informatics tool designed to improve health outcomes for patients with complex multimorbidity, using a team- and data-driven patient grouping approach. In this formative pilot, we found that among participating VHA teams, the unique features of the tool, including the application of population segmentation models to a clinical team’s patient panel, automatic identification of data-driven “care step” suggestions, and workflows that enabled multidisciplinary input into patient care, were perceived as useful and desirable during both the user design and preliminary testing. In the preliminary testing with frontline teams, general primary care teams with variation in staffing, site context, and prior panel management experience reported high VET-PATHS acceptability and utility during facilitated team meetings, and were able to review 44% to 96% of their empaneled patients in the tool over 4 to 8 brief team meetings, planning a change in care for 1 in every 3 patients reviewed, such as new vaccines, laboratory tests, or evidence-based consults. In contrast, focused “specialized” primary care teams had lower acceptability and feasibility. While individual care steps directly prompted by the tool had variable utility, participating teams enacted new care changes not directly from the tool and perceived value in the multidisciplinary discussion and review process, supporting that both the tool use context and content were important. We observed favorable trends in most utilization outcomes for pilot team patients, though this feasibility study was not designed nor powered to evaluate for statistically significant changes in these outcomes. This pilot covers the user-centered design and preliminary testing of a VHA-specific tool developed and evaluated via a learning health system approach (applying health system data patterns to inform current patient care) [48].

Based on the observations and feedback from this pilot, we plan to focus future testing of the tool on VHA general primary care teams with heterogeneous Veteran patient panels, which care for almost 90% of patients at high risk in the VHA [49]. We are currently revising the tool with the clinical group, care step, and functionality updates, including contemporary data-driven multimorbidity groups, expanded and alternative care steps based on perceived relevance and clinical practice guidelines, increased comprehensive care summarized data based on team feedback, and greater explanation of data sources and methodology within the tool. Given variable uptake of some care steps, prompts for shared decision-making and patient interface with care recommendations may increase the usefulness of care steps that require more complex discussion with patients. Furthermore, we note that most of the patients included in the tool are being seen by PCPs regularly. Most “due” care steps represent interventions indicated for a long time, with possible prior recommendation by providers, but not otherwise received. Therefore, even modest uptake of care suggested by the tool among these patients, if translated using a population health perspective, could have significance. For example, 5 of 27 (18.5%) patients “due” for hepatitis B vaccination in the substance use group received it, following tool use. Among the 819,000 Veterans enrolled in the VHA with a prior substance use disorder diagnosis still susceptible to hepatitis B infection [50], similar proportions would lead to over 151,000 additional Veterans immunized.

### Comparison to Prior Work

Patients with multimorbidity are increasingly prevalent and costly for health systems to care for [1,2,4]. In the face of primary care staffing shortages [51], there is a growing mismatch between available clinical resources and the effort needed to provide personalized and effective care to patients with multimorbidity [7,8]. Tools to facilitate care planning could improve the efficiency and effectiveness of care for this population, but barriers to use exist. A 2018 survey of 1804 VHA PACT clinicians found that informatics tool use for complex patients was impeded by feelings of being “overwhelmed” by the amount and variety of information displayed and lack of guidance from tools on what patient needs to prioritize [14]. In VHA, as in other health systems, registries of patients predicted to have high risk for adverse events are provided to teams but do not help clinicians identify actions to take to lower risk [15]. Two main takeaways from this work are relevant to other health systems. First, this pilot presents a novel bridging of validated population-level prediction and population segmentation analytics [15,36] with individualized clinical care recommendations in a team-based, primary care intervention [15,27]. Other health systems can use similar methods to segment their patient populations and incorporate the results into their panel management tools or apply similar workflows in their primary care settings. Second, our development and evaluation process is a useful example of tool co-design with frontline health care staff within a learning health system. Few detailed examples spanning early development to real-world testing for digital innovations in primary care have been published [52].

### Strengths and Limitations

There are strengths and limitations to this study. User-centered design and acceptability interviews were conducted with interested, participating frontline clinical staff in multiple team roles in VHA medical centers, following the principles of co-design with target users. This process can be replicated outside the VHA, but the specific feedback given by VHA users may not generalize to non-VHA clinical settings. Teams within VHA, however, participated from a variety of clinic settings, staffing levels, and prior panel management experience. Pilot teams were supported by trained facilitators, which may not be pragmatic to offer throughout health systems. However, brief training and “champion user” facilitation are commonly used approaches by health systems helping health care teams adopt new technology tools [53]. Additionally, as previously described, facilitators had key roles in training at the onset, followed by smaller technical assistance roles for subsequent meetings that could be filled by lower-resource methods (eg, on-demand assistance services or more in-depth reference materials). VET-PATHS is focused on Veterans in primary care settings at high risk of hospitalization, so results may not fully generalize to patient populations in other health care settings. While we used several methods to check the accuracy and validity of facilitator observations, we acknowledge that this data could be susceptible to confirmation bias, although we attempted to mitigate this via structured collection templates and dually staffed observations. Given these limitations, the next crucial step is a full, randomized trial of the effectiveness of using VET-PATHS in broader, real-world primary care settings with evaluation of patient and clinician outcomes. Testing tools like VET-PATHS in real-world settings will also build evidence for how panel management tools for patients with multimorbidity can be best integrated into typical primary care team workflows. Finally, we do not have follow-up data on the subsequent patient experience or shared decision-making that will be integral to future capture of the full impact of personalized care planning for primary care patients at high risk.

### Conclusions and Future Directions

VET-PATHS, developed through user-centered design and testing, was feasible and acceptable for participating primary care teams in the VHA to use to develop new care plans for their patients with complex multimorbidity and led to several documented clinical impacts, including the delivery of new evidence-based care to patients. VET-PATHS demonstrates a tool and workflow linking population-segmentation analytics and clinical care, a novel preliminary study of an approach for teams managing complex patients at high risk. In our learning health system, multimorbidity group and care step information within VET-PATHS is being iteratively updated, in response to changing EHR data patterns and clinician feedback. Future directions include a planned full effectiveness trial of VET-PATHS with primary care teams and ongoing study of the impact of iterative updates to VET-PATHS.

## Supplementary material

10.2196/75081 Multimedia Appendix 1 Tool snapshots.

10.2196/75081 Multimedia Appendix 2 Chronic conditions used to define multimorbidity groups.

10.2196/75081 Multimedia Appendix 3 User-centered design phase results.

10.2196/75081 Multimedia Appendix 4 Per–care step uptake and activity by multimorbidity group (4 of 6 groups with active care steps at the time of the pilot).

## Abbreviations

CA: clinical associate
CAN: Care Assessment Need
CBOC: community-based outpatient clinic
EHR: electronic health record
MSA: medical support administrator
PACT: Patient Aligned Care Teams
PCP: primary care clinician
RN: registered nurse
SUS: System Usability Scale
VAMC: VHA medical center–affiliated clinic
VET-PATHS: Veteran Panel Management Tool for High-Risk Subgroups
VHA: Veterans Health Administration
