# Supplementary material for: A Novel Primary Care Planning Informatics Tool Informed by Data-Driven Multimorbidity Grouping: User-Centered Design and Feasibility Testing
Source: JMIR Form Res. 2025 Dec 4;9:e75081. doi: 10.2196/75081 (PMC12677874; doi:10.2196/75081)
Supplement: Multimedia Appendix 1 [file formative-v9-e75081-s001.docx]

**Appendix 1. Tool snapshots**

**VET-PATHS, Screenshot 1 (below)**: Front page, showing tool link (in advanced prototype, the link is called “CAN by Comorbidity” as a descriptive title) embedded within other targeted panel management tools in the PCAS home screen (a website within VA Intranet). Teams can review whole list of high-risk patients, or drop downs offer ability to sort by Multimorbidity Group or only look at one Group at a time.


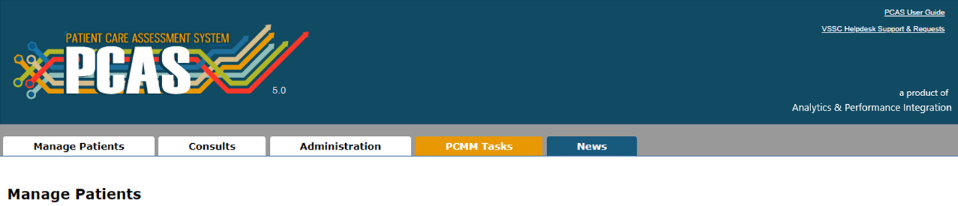

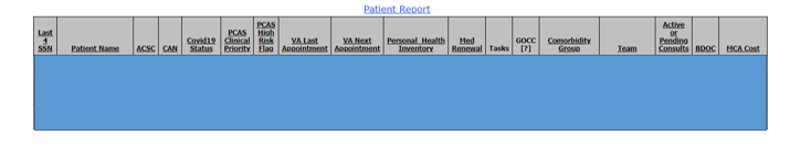

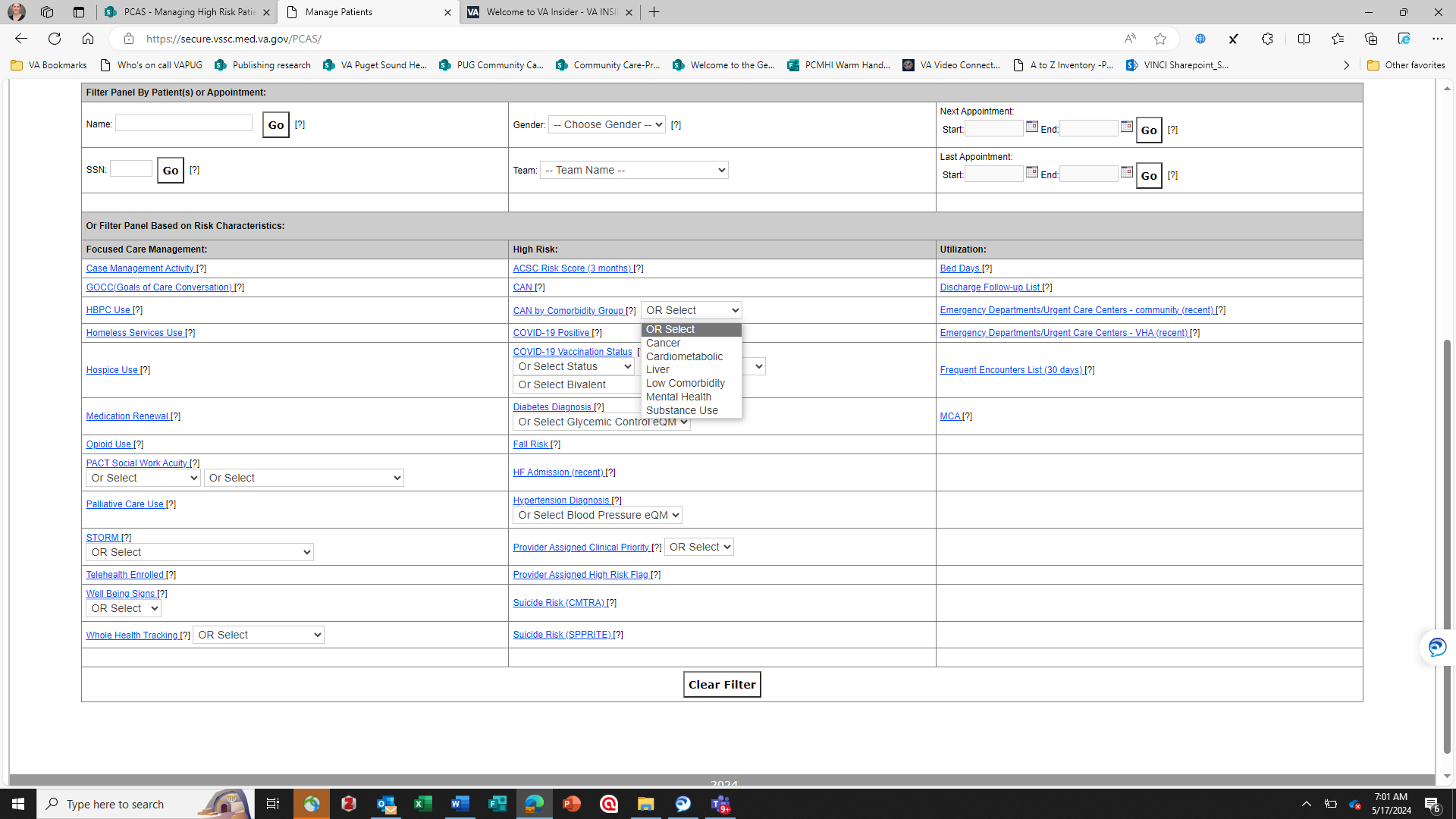


**VET-PATHS**

**All high-risk patient in VET-PATHS “list” view**

**VET-PATHS, Screenshot 2 (below)**: Individual patient-level page profile view. Shows **specific patient chronic condition diagnoses**, within the overall cluster of comorbidities within a **high-risk, Multimorbidity Group**. The tool provides recommended, group-tailored, data-drive **care steps** that teams can consider, that show if a patient has received guideline-recommended care and/or care that is associated with reduced hospitalization outcomes from group-specific statistical models.


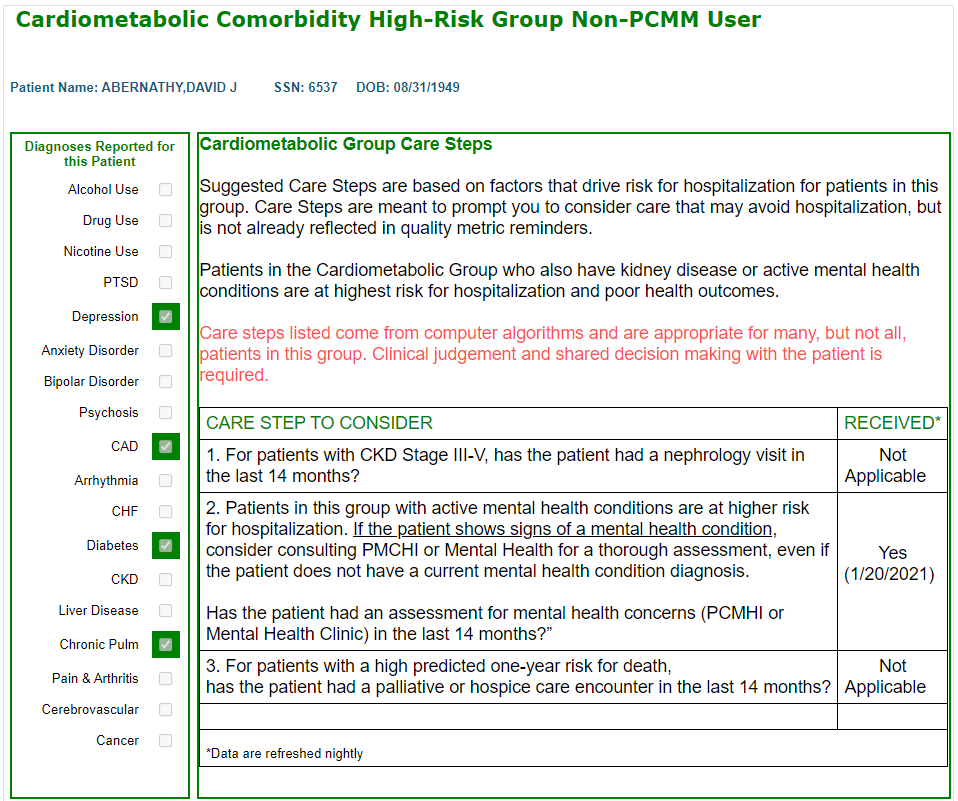


**Care steps & indicator of receipt for the individual patient.**

**“Snapshot” of individual patient diagnoses, within those possible that define the Multimorbidity Group**

**VET-PATHS, Screenshot 3 (below)**: Individual patient-level page profile view, if user scrolls down to page 2 of the profile. Provides teams that are interested with **optional education and insights on the Multimorbidity Group**, including from clinical lessons based on findings from latent data models, and overview of the clusters of diseases in the group.


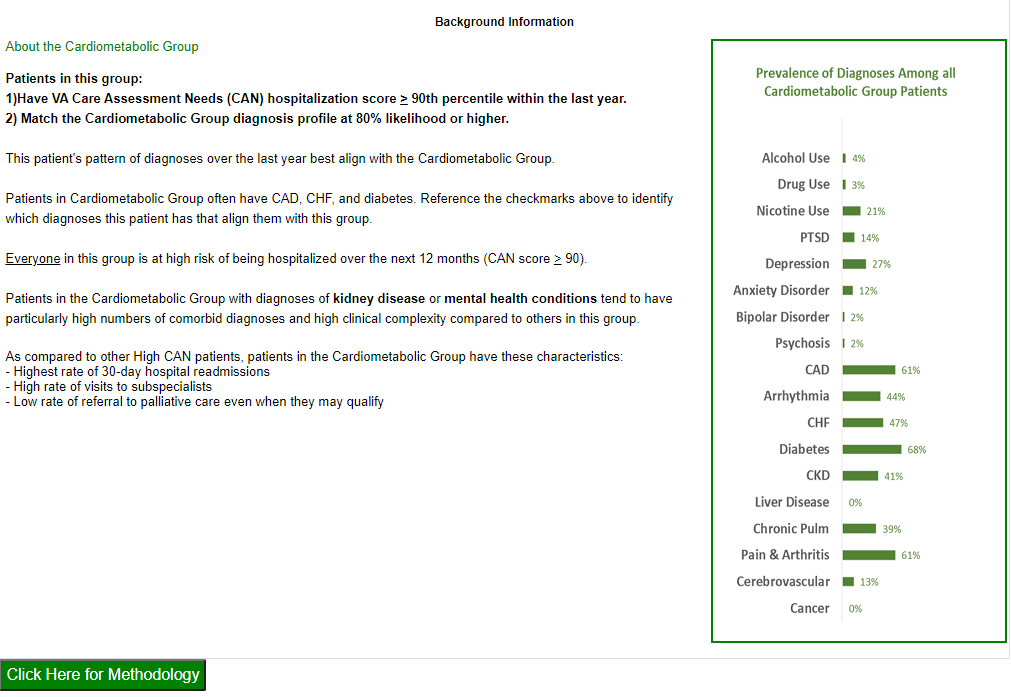


**Optional education on the specific Multimorbidity Group**
