# Supplementary material for: A Novel Primary Care Planning Informatics Tool Informed by Data-Driven Multimorbidity Grouping: User-Centered Design and Feasibility Testing
Source: JMIR Form Res. 2025 Dec 4;9:e75081. doi: 10.2196/75081 (PMC12677874; doi:10.2196/75081)
Supplement: Multimedia Appendix 2 [file formative-v9-e75081-s002.docx]

**Appendix 2. Chronic conditions used to define multimorbidity groups**

- Alcohol use disorder
- Anemia
- Anxiety
- Arthritis
- Asthma
- Bipolar disorder
- Congestive heart failure
- Chronic obstructive pulmonary disease
- Cancer
- Cardiac arrhythmias
- Cerebrovascular
- Coronary artery disease
- Dementias
- Depressive disorders
- Diabetes
- Gastrointestinal diseases
- Headache
- Hepatitis/Liver disease
- Hyperlipidemia
- Hypertension
- Kidney disease
- Low back pain
- Neuropathy
- Posttraumatic stress disorder
- Peripheral vascular disease
- Prostate/Prostatic Hyperplasia
- Psychosis
- Sleep wake disorders
- Substance use disorders
- Thyroid disorders
- Tobacco
