# Supplementary material for: A Novel Primary Care Planning Informatics Tool Informed by Data-Driven Multimorbidity Grouping: User-Centered Design and Feasibility Testing
Source: JMIR Form Res. 2025 Dec 4;9:e75081. doi: 10.2196/75081 (PMC12677874; doi:10.2196/75081)
Supplement: Multimedia Appendix 3 [file formative-v9-e75081-s003.docx]

**Appendix 3. User centered design phase results.**

| **Round** | **Current user experience** | **Requested/completed updates** |
| --- | --- | --- |
| 1  (RN, n=6, paper/static prototypes, 2019) | Useful: Appreciated perspectives on different subgroups. Most high usefulness to PCPs, not RNs. Helpful for group management, follow-up care, tracking.  Usability: Text largely understandable, usable, other feedback led to updates at right. Desirable: Value-add for proactive panel management, for care transitions, for new patients, to identify patients needing intensive follow-up.  Credible: Seen as credible to use of known risk-prediction score/diagnoses, planned integration into an existing web-based panel management site. | Minimize redundancy, clarifying when information applied to group vs. individual, implied associations between color of images and text. Graphics and text updated and streamlined (i.e., methodology descriptions removed) throughout based on feedback. Added pop-up text boxes instead of embedded text to reduce text burden, added user-requested columns and views to patient grid view.  Interview guide reduced, tailored to next phase. |
| 2  (RN & PCP, n=8, electronic visuals, 2019) | Useful: Contexts for optimal usefulness noted as preparing for visit, engaging in panel management for a group of patients, providing feedback to patients on why care steps suggested.  Usability: Led to updates at right.  Desirable: More value if non-redundant with information in other tools, integrated with current EHR, current, regularly update, and care steps non-redundant with other reminders.  Credible: Clarity on how frequently updated would help. | Explanatory text boxes standardized, updates to format, graphics, and text throughout (e.g., presenting of individual vs. group data). Subgroups renamed. On individual patient pages, figures re-organized to prioritize individual patient data on top, with group information at bottom as reference. Overall tool renamed to distinguish from other available tools. |
| 3  (PCPs, n=3, interactive web-based prototype, 2021) | Usability/Useful: Some users wanted increased data linked in, such as baseline and current lab values for patients. More usable if integrated into EHR, some limitation in usability and usefulness due to current interface formatting. Request for grid overview for all patients. Felt useful to have overview of similar patients, with existing filters (e.g., CAN scores) presented.  Desirable: Care steps felt to be helpful for presenting potential “misses” in care not otherwise considered.  Credible: Increased transparency about origin of care step data requested. | Formatting updated, added details on data sources, wording updates on care steps and text within tool. |
