# Supplementary material for: A Novel Primary Care Planning Informatics Tool Informed by Data-Driven Multimorbidity Grouping: User-Centered Design and Feasibility Testing
Source: JMIR Form Res. 2025 Dec 4;9:e75081. doi: 10.2196/75081 (PMC12677874; doi:10.2196/75081)
Supplement: Multimedia Appendix 4 [file formative-v9-e75081-s004.docx]

**Appendix 4 – Per-care step uptake and activity by multimorbidity group (4 of 6 groups with active care steps at time of pilot).**

| **Cardio-metabolic** | **Total care steps** | **Due** | | **Discussed** | | **Due &**  **Discussed** | **No action on due** | **Action Intended** | | **Action**  **Ordered / Placed** | | **Action**  **Occurred** |
| --- | --- | --- | --- | --- | --- | --- | --- | --- | --- | --- | --- | --- |
| **Mental health visit in overdue or MH diagnosis patients** | 144 | 72% (103/  144) | | 73% (105/144) | | 57% (82/144) | 85% (70/ 82) | 15% (12/82) | | 17%  (2/12) | | 17%  (2/12) |
| **Nephrology referral in CKD stage 3 or higher** | 144 | 8% (12/ 144) | | 48% (69/144) | | 8% (12/144) | 83% (10/ 12) | 17%  (2/12) | | 100%  (2/2) | | 100%  (2/2) |
| **Palliative care referral** | 144 | 60% (87/ 144) | | 65% (94/144) | | 47% (67/144) | 84% (56/ 67) | 16%  (11/67) | | 18%  (2/11) | | 9%  (1/11) |
| **Total care steps** | | ***47% (202/***  ***432)*** | | ***62% (268/432)*** | | ***37% (161/432)*** | ***84% (136/***  ***161)*** | ***15% (25/161)*** | | ***24%***  ***(6/25)*** | | ***20%***  ***(5/25)*** |
| **Why no action, if due and discussed? (n=136 care steps)** | | | | | | | | | | | | |
| **Team thought care not appropriate**  46% (62/136) | | | | | **Team thought care appropriate, but other reason for no action**  24% (32/136) | | | | **Unable to assess appropriateness and no action taken**  31% (42/136) | | | |
| *Provider judgement* | | | 53%  (33/62) | | *Patient already scheduled for care* | | 38%  (12/32) | | *New patient to team, need to assess first* | | 2%  (1/42) | |
| *Data wrong* | | | 3%  (2/62) | | *Data wrong* | | 13%  (4/32) | | *Not on panel* | | 7%  (3/42) | |
| *Patient doing well, thought not needed* | | | 44%  (27/62) | | *Previously declined* | | 47%  (15/32) | | *Provider judgement, didn’t state why* | | 7%  (3/42) | |
|  | | |  | | *Reassessment needed first* | | 8%  (1/32) | | *Reassessment needed first* | | 2%  (1/42) | |
|  | | |  | |  | |  | | *Unknown* | | 81%  (34/42) | |

| **Mental Health** | **Total Care Steps** | | **Due** | | **Discussed** | **Due &**  **Discussed** | | **No action on due** | | **Action Intended** | **Action**  **Ordered / Placed** | **Action**  **Occurred** |
| --- | --- | --- | --- | --- | --- | --- | --- | --- | --- | --- | --- | --- |
| **Mental health visit in overdue or MH diagnosed patients** | 56 | | 48%  (27/ 56) | | 84%  (47/56) | 46%  (26/56) | | 78%  (21/ 27) | | 19%  (5/26) | 60%  (3/5) | 60%  (3/5) |
| **Diabetes screening in non-diabetics** | 56 | | 16%  (9/ 56) | | 86%  (48/56) | 16%  (9/56) | | 56%  (5/9) | | 44%  (4/9) | 75%  (3/4) | 75%  (3/4) |
| **Lipid testing in due patients** | 56 | | 23%  (13/ 56) | | 80%  (45/56) | 23%  (13/56) | | 77%  (10/ 13) | | 23%  (3/13) | 100%  (3/3) | 100%  (3/3) |
| **Diabetes manage-ment class in diabetics** | 56 | | 41%  (23/ 56) | | 86%  (48/56) | 41%  (23/56) | | 96%  (22/ 23) | | 4%  (1/23) | 0%  (0/1) | 0%  (0/1) |
| **Secure messaging enrollment in unenrolled** | 56 | | 48%  (27/ 56) | | 84%  (47/56) | 48%  (27/56) | | 74%  (20/ 27) | | 26%  (7/27) | 29%  (2/7) | 14%  (1/7) |
| **Total care steps** | | | ***35% (99/***  ***280)*** | | ***84% (235/280)*** | ***35% (98/280)*** | | ***79%***  ***(78/ 99)*** | | ***20%***  ***(20/98)*** | ***55%***  ***(11/20)*** | ***50%***  ***(10/20)*** |
| **Why no action, if due and discussed? (n=78)** | | | | | | | | | | | | |
| **Team thought care not appropriate**  36% (28/78) | | | | **Team thought care appropriate, but other reason for no action**  35% (27/78) | | | | | **Unable to assess appropriateness and no action taken**  29% (23/78) | | | |
| *Patient already scheduled for care* | | 7%  (2/28) | | *Patient already scheduled for care* | | | 43%  (13/27) | | *Other* | | 9%  (2/23) | |
| *Data wrong* | | 14%  (4/28) | | *Data wrong* | | | 30%  (9/27) | | *Unknown / team didn’t specify* | | 87%  (20/23) | |
| *Patient declined previously* | | 4%  (1/28) | | *Patient declined previously* | | | 10%  (3/27) | | *Provider judgement, didn’t state why* | | 4%  (1/23) | |
| *Provider judgement* | | 54%  (15/28) | | *Other* | | | 7%  (2/27) | |  | |  | |
| *Patient doing well* | | 21%  (6/28) | |  | | |  | |  | |  | |

| **Substance Use** | **Total Care Steps** | | **Due** | **Discussed** | **Due &**  **Discussed** | | **No action on due** | | **Action**  **Intended** | **Action**  **Ordered / Placed** | | **Action**  **Occurred** |
| --- | --- | --- | --- | --- | --- | --- | --- | --- | --- | --- | --- | --- |
| **Mental health visit in overdue or MH diagnosed patients** | 86 | | 28%  (24/ 86) | 47%  (40/86) | 7%  (6/86) | | 100%  (6/6) | | 0%  (0/6) | 0%  (0/6) | | 0%  (0/6) |
| **Secure messaging enrollment in unenrolled** | 86 | | 69%  (59/ 86) | 41%  (35/86) | 29%  (25/86) | | 68%  (17/25) | | 32%  (8/25) | 88%  (7/8) | | 13%  (1/8) |
| **Offer video visit in unenrolled** | 86 | | 58%  (50/ 86) | 48%  (41/86) | 28%  (24/86) | | 92%  (22/24) | | 8%  (2/24) | 50%  (1/2) | | 0%  (0/2) |
| **Hep B vaccine in non-immune or unvaccinated** | 86 | | 62%  (53/ 86) | 43%  (37/86) | 31%  (27/86) | | 26%  (7/27) | | 74% (20/27) | 35%  (7/20) | | 25%  (5/20) |
| **Total care steps** | | | ***54%***  ***(186/***  ***344)*** | ***44%***  ***(153/344)*** | ***24%***  ***(82/344)*** | | ***63%***  ***(52/82)*** | | ***37%***  ***(30/82)*** | ***42%***  ***(15/36)*** | | ***17%***  ***(6/36)*** |
| **Why no action, if due and discussed? (n=52)** | | | | | | | | | | | | |
| **Team thought care not appropriate**  *25% (13/52)* | | | | **Team thought care appropriate, but other reason for no action**  *46% (24/52)* | | | | **Unable to assess appropriateness and no action taken**  29% (15/52) | | | | |
| *Provider judgement* | | *92%*  *(12/13)* | | *Patient already scheduled for care* | | *58%*  *(14/24)* | | *Unknown / team didn’t specify* | | | 93%  (14/15) | |
| *Other* | | *8%*  *(1/13)* | | *Data wrong* | | *29%*  *(7/24)* | | *Patient doing well* | | | 7%  (1/15) | |
|  | |  | | *Patient declined previously* | | *13%*  *(3/24)* | |  | | |  | |
|  | |  | | *Other* | | *4%*  *(1/24)* | |  | | |  | |

| **Liver** | **Total Care Steps** | | **Due** | **Discussed** | **Due &**  **Discussed** | | **No action on due** | | **Action**  **Intended** | **Action**  **Ordered / Placed** | | **Action**  **Occurred** |
| --- | --- | --- | --- | --- | --- | --- | --- | --- | --- | --- | --- | --- |
| **Referral to home-based primary care** | 63 | | 86%  (54/ 63) | 100%  (63/63) | 86%  (54/63) | | 98%  (53/ 54) | | 2%  (1/54) | 0%  (0/1) | | 0%  (0/1) |
| **Hep B vaccine in non-immune or un-vaccinated** | 63 | | 27%  (17/ 63) | 100%  (63/63) | 27%  (17/63) | | 76%  (13/ 17) | | 24%  (4/17) | 50%  (2/4) | | 50%  (2/4) |
| **Nephrology referral in CKD stage 3 or higher** | 63 | | 6%  (4/ 63) | 71%  (45/63) | 6%  (4/63) | | 100%  (4/4) | | 0%  (0/4) | 0%  (0/0) | | 0%  (0/0) |
| **Palliative care referral** | 63 | | 54%  (34/ 63) | 87%  (55/63) | 54%  (34/63) | | 79%  (27/ 34) | | 21%  (7/34) | 14%  (1/7) | | 14%  (1/7) |
| **Total care steps** | | | ***43%***  ***(109/***  ***252)*** | ***90%***  ***(226/252)*** | ***43%***  ***(109/252)*** | | ***89%***  ***(97/ 109)*** | | ***11%***  ***(12/109)*** | ***25%***  ***(3/12)*** | | ***25%***  ***(3/12)*** |
| **Why no action, if due and discussed? (n=97)** | | | | | | | | | | | | |
| **Team thought care not appropriate**  25% (24/97) | | | | **Team thought care appropriate, but other reason for no action**  14% (14/97) | | | | **Unable to assess appropriateness and no action taken**  61% (59/97) | | | | |
| *Provider judgement* | | *100%*  *(24/24)* | | *Data wrong* | | 71%  (10/14) | | *Unknown / team didn’t specify* | | | 58%  (34/59) | |
|  | |  | | *Care already scheduled* | | 7%  (1/14) | | *Patient doing well* | | | 17%  (10/59) | |
|  | |  | | *Assess before action* | | 7%  (1/14) | | *Reassessment needed* | | | 10%  (6/59) | |
|  | |  | | *Declined previously* | | 7%  (1/14) | | *Not on panel* | | | 8%  (5/59) | |
|  | |  | | *Patient doing well* | | 7%  (1/14) | | *Provider judgement, didn’t state why* | | | 7%  (4/59) | |
